# A transcriptome-wide association study based on 27 tissues identifies 106 genes potentially relevant for disease pathology in age-related macular degeneration

**DOI:** 10.1038/s41598-020-58510-9

**Published:** 2020-01-31

**Authors:** Tobias Strunz, Susette Lauwen, Christina Kiel, International AMD Genomics Consortium (IAMDGC), Lars G. Fritsche, Wilmar Igl, Jessica N. Cooke Bailey, Felix Grassmann, Sebanti Sengupta, Jennifer L. Bragg-Gresham, Kathryn P. Burdon, Scott J. Hebbring, Cindy Wen, Mathias Gorski, Ivana K. Kim, David Cho, Donald Zack, Eric Souied, Hendrik P. N. Scholl, Elisa Bala, Kristine E. Lee, David J. Hunter, Rebecca J. Sardell, Paul Mitchell, Joanna E. Merriam, Valentina Cipriani, Joshua D. Hoffman, Tina Schick, Yara T. E. Lechanteur, Robyn H. Guymer, Matthew P. Johnson, Yingda Jiang, Chloe M. Stanton, Gabriëlle H. S. Buitendijk, Xiaowei Zhan, Alan M. Kwong, Alexis Boleda, Matthew Brooks, Linn Gieser, Rinki Ratnapriya, Kari E. Branham, Johanna R. Foerster, John R. Heckenlively, Mohammad I. Othman, Brendan J. Vote, Helena Hai Liang, Emmanuelle Souzeau, Ian L. McAllister, Timothy Isaacs, Janette Hall, Stewart Lake, David A. Mackey, Ian J. Constable, Jamie E. Craig, Terrie E. Kitchner, Zhenglin Yang, Zhiguang Su, Hongrong Luo, Daniel Chen, Hong Ouyang, Ken Flagg, Danni Lin, Guanping Mao, Henry Ferreyra, Klaus Stark, Claudia N. von Strachwitz, Armin Wolf, Caroline Brandl, Guenther Rudolph, Matthias Olden, Margaux A. Morrison, Denise J. Morgan, Matthew Schu, Jeeyun Ahn, Giuliana Silvestri, Evangelia E. Tsironi, Kyu Hyung Park, Lindsay A. Farrer, Anton Orlin, Alexander Brucker, Mingyao Li, Christine Curcio, Saddek Mohand-Saïd, José-Alain Sahel, Isabelle Audo, Mustapha Benchaboune, Angela J. Cree, Christina A. Rennie, Srinivas V. Goverdhan, Michelle Grunin, Shira Hagbi-Levi, Peter Campochiaro, Nicholas Katsanis, Frank G. Holz, Frédéric Blond, Hélène Blanché, Jean-François Deleuze, Robert P. Igo, Barbara Truitt, Neal S. Peachey, Stacy M. Meuer, Chelsea E. Myers, Emily L. Moore, Ronald Klein, Michael A. Hauser, Eric A. Postel, Monique D. Courtenay, Stephen G. Schwartz, Jaclyn L. Kovach, William K. Scott, Gerald Liew, Ava G. Tfan, Bamini Gopinath, John C. Merriam, R. Theodore Smith, Jane C. Khan, Humma Shahid, Anthony T. Moore, J. Allie McGrath, Reneé Laux, Milam A. Brantley, Anita Agarwal, Lebriz Ersoy, Albert Caramoy, Thomas Langmann, Nicole T. M. Saksens, Eiko K. de Jong, Carel B. Hoyng, Melinda S. Cain, Andrea J. Richardson, Tammy M. Martin, John Blangero, Daniel E. Weeks, Bal Dhillon, Cornelia M. van Duijn, Kimberly F. Doheny, Jane Romm, Caroline C. W. Klaver, Caroline Hayward, Michael B. Gorin, Michael L. Klein, Paul N. Baird, Anneke I. den Hollander, Sascha Fauser, John R. W. Yates, Rando Allikmets, Jie Jin Wang, Debra A. Schaumberg, Barbara E. K. Klein, Stephanie A. Hagstrom, Itay Chowers, Andrew J. Lotery, Thierry Léveillard, Kang Zhang, Murray H. Brilliant, Alex W. Hewitt, Anand Swaroop, Emily Y. Chew, Margaret A. Pericak-Vance, Margaret DeAngelis, Dwight Stambolian, Jonathan L. Haines, Sudha K. Iyengar, Bernhard H. F. Weber, Gonçalo R. Abecasis, Iris M. Heid, Anneke den Hollander, Bernhard H. F. Weber

**Affiliations:** 1 Institute of Human Genetics, University of Regensburg, Regensburg, Germany; 2 Department of Ophthalmology, Donders Institute for Brain, Cognition and Behaviour, Radboud university medical center, Nijmegen, The Netherlands; 3 Department of Human Genetics, Donders Institute for Brain, Cognition and Behaviour, Radboud university medical center, Nijmegen, The Netherlands; 4 Institute for Human Genomics, University of Miami Miller School of Medicine, Miami, USA; 5 Center for Statistical Genetics, Department of Biostatistics, University of Michigan, Ann Arbor, MI 48109 USA; 6 Department of Genetic Epidemiology, University of Regensburg, Regensburg, Germany; 7 Department of Epidemiology and Biostatistics, Case Western Reserve University School of Medicine, 2103 Cornell Rd, Cleveland, OH 44106 USA; 8 Institute of Human Genetics, University of Regensburg, Regensburg, Germany; 9 Kidney Epidemiology and Cost Center, Department of Biostatistics, Department of Internal Medicine - Nephrology, University of Michigan, Ann Arbor, MI 48109 USA; 10 School of Medicine, Menzies Research Institute Tasmania, University of Tasmania, Hobart, Tasmania Australia; 11 Center for Human Genetics, Marshfield Clinic Research Foundation, 1000N. Oak Ave, Marshfield, WI 54449 USA; 12 Department of Ophthalmology, University of California San Diego and VA San Diego Health System, La Jolla, California, 92093 USA; 13 Retina Service, Massachusetts Eye and Ear, Department of Ophthalmology Harvard Medical School, Boston, MA USA; 14 Department of Ophthalmology, Perelman School of Medicine, University of Pennsylvania, Philadelphia, USA; 15 Department of Ophthalmology, Wilmer Eye Institute - Johns Hopkins University School of Medicine - 400 North Broadway, Smith Building -, Baltimore, Maryland 21287 USA; 16 Department of Molecular Biology and Genetics - Johns Hopkins University School of Medicine - 400 North Broadway, Smith Building -, Baltimore, Maryland 21287 USA; 17 Department of Neuroscience - Johns Hopkins University School of Medicine - 400 North Broadway, Smith Building -, Baltimore, Maryland 21287 USA; 18 Institute of Genetic Medicine - Johns Hopkins University School of Medicine - 400 North Broadway, Smith Building -, Baltimore, Maryland 21287 USA; 19 Institue de la Vision, Université Pierre et Marie Curie, 17 rue Moreau, Paris, France; 20 Hôpital Intercommunal de Créteil, Hôpital Henri Mondor - Université Paris Est Créteil, Créteil, France; 21 University of Bonn - Department of Ophthalmology - Ernst-Abbe-Str. 2, D-53127 Bonn, Germany; 22 Louis Stokes Cleveland VA Medical Center, 10701 East Boulevard, Cleveland, OH 44106 USA; 23 Department of Ophthalmology and Visual Sciences, University of Wisconsin, Madison, WI USA; 24 Department of Epidemiology, Harvard School of Public Health, Boston, USA; 25 Department of Nutrition, Harvard School of Public Health, Boston, USA; 26 John P. Hussman Institute for Human Genomics, Miller School of Medicine, University of Miami, Miami, Florida United States; 27 Centre for Vision Research, Department of Ophthalmology and Westmead Millennium Institute for Medical Research, University of Sydney, Sydney, Australia; 28 Department of Ophthalmology Columbia University, New York, NY USA; 29 UCL Institute of Ophthalmology, University College London, London, EC1V 9EL UK; 30 Moorfields Eye Hospital, London, EC1V 2PD UK; 31 Center for Human Genetics Research, Vanderbilt University Medical Center, Nashville, Tennessee United States; 32 University Hospital of Cologne, Department of Ophthalmology, Kerpener Str. 62, 50924 Cologne, Germany; 33 Department of Ophthalmology, Radboud University Medical Centre, Nijmegen, the Netherlands; 34 Centre for Eye Research Australia, University of Melbourne, Royal Victorian Eye and Ear Hospital, East Melbourne, Victoria, 3000 Australia; 35 South Texas Diabetes and Obesity Institute, University of Texas Rio Grande Valley School of Medicine, Brownsville, TX 78520 USA; 36 Department of Biostatistics, Graduate School of Public Health, University of Pittsburgh, Pittsburgh, PA 15261 USA; 37 MRC Human Genetics Unit, Institute of Genetics and Molecular Medicine, University of Edinburgh, EH4 2XU Scotland, UK; 38 Department of Ophthalmology, Erasmus Medical Center, Rotterdam, PO box 2040, 3000CA The Netherlands; 39 Department of Epidemiology, Erasmus Medical Center, Rotterdam, PO box 2040, 3000CA The Netherlands; 40 Quantitative Biomedical Research Center, Department of Clinical Science, University of Texas Southwestern Medical Center, 5323 Harry Hines Boulevard, Dallas, TX 75390 USA; 41 Center for the Genetics of Host Defense, University of Texas Southwestern Medical Center, 5323 Harry Hines Boulevard, Dallas, TX 75390‐8505 USA; 42 Neurobiology Neurodegeneration & Repair Laboratory (N-NRL), National Eye Institute, National Institutes of Health, Bethesda, MD 20892 USA; 43 Department of Ophthalmology and Visual Sciences, University of Michigan, Kellogg Eye Center, Ann Arbor, MI 48105 USA; 44 Department of Ophthalmology, Flinders Medical Centre, Flinders University, Adelaide, South Australia Australia; 45 Centre for Ophthalmology and Visual Science, Lions Eye Institute, University of Western Australia, Perth, Western Australia Australia; 46 Sichuan Provincial Key Laboratory for Human Disease Gene Study, Hospital of the University of Electronic Science and Technology of China and Sichuan Provincial People’s Hospital, Chengdu, China; 47 Sichuan Translational Medicine Hospital, Chinese Academy of Sciences, Chengdu, China; 48 Molecular Medicine Research Center, State Key Laboratory of Biotherapy, West China Hospital, Sichuan University, Sichuan, 610041 China; 49 EyeCentre Southwest Stuttgart, Stuttgart, Germany; 50 University Eye Clinic, Ludwig-Maximilians-University Munich, Munich, Germany; 51 Department of Ophthalmology, University Hospital Regensburg, Regensburg, Germany; 52 Department of Ophthalmology and Visual Sciences, University of Utah, Salt Lake City, UT USA; 53 Department of Medicine (Biomedical Genetics), Boston University Schools of Medicine and Public Health, Boston, MA USA; 54 Department of Ophthalmology, Boston University Schools of Medicine and Public Health, Boston, MA USA; 55 Department of Neurology, Boston University Schools of Medicine and Public Health, Boston, MA USA; 56 Department of Epidemiology, Boston University Schools of Medicine and Public Health, Boston, MA USA; 57 Department of Biostatistics, Boston University Schools of Medicine and Public Health, Boston, MA USA; 58 Department of Ophthalmology, Seoul Metropolitan Government Seoul National University Boramae Medical Center, Seoul, Republic of Korea; 59 Centre for Experimental Medicine, Queen’s University, Belfast, UK; 60 Department of Ophthalmology, University of Thessaly, School of Medicine, Larissa, Greece; 61 Department of Ophthalmology, Seoul National University Bundang Hospital, Seongnam, Republic of Korea; 62 Department of Ophthalmology, Weill Cornell Medical College, New York, NY USA; 63 Scheie Eye Institute, Department of Ophthalmology, University of Pennsylvania Perelman School of Medicine, Philadelphia, PA 19104 USA; 64 Department of Biostatistics and Epidemiology University of Pennsylvania Perelman School of Medicine, Philadelphia, PA 19104 USA; 65 Department of Ophthalmology, The University of Alabama at Birmingham 1670 University Boulevard VH 360, Birmingham, AL 35294-0019 UK; 66 INSERM, U968, Paris, F-75012 France; 67 UPMC Univ Paris 06, UMR_S 968, Institut de la Vision, Department of Genetics, Paris, F-75012 France; 68 CNRS, UMR_7210, Paris, F-75012 France; 69 Centre Hospitalier National d’Ophtalmologie des Quinze-Vingts, INSERM-DHOS CIC 503, Paris, F-75012 France; 70 Fondation Ophtalmologique Adolphe de Rothschild, Paris, F-75019 France; 71 Institute of Ophthalmology, University College of London, London, WC1E 6BT UK; 72 Académie des Sciences–Institut de France, Paris, F-75006 France; 73 Department of Molecular Genetics, Institute of Ophthalmology, London, UK; 74 Clinical and Experimental Sciences, Faculty of Medicine, University of Southampton, Southampton, UK; 75 University Hospital Southampton, Southampton, UK; 76 Department of Ophthalmology, Hadassah Hebrew University Medical Center, Jerusalem, Israel; 77 Center for Human Disease Modeling, Duke University, Durham, USA; 78 Department of Cell Biology, Duke University, Durham, USA; 79 Department of Pediatrics, Duke University, Durham, USA; 80 CEPH Fondation Jean Dausset 27 rue Juliette Dodu, 75010 Paris, France; 81 CEA – IG – Centre National de Génotypage 2 rue Gaston Crémieux, 91057 Evry Cédex, France; 82 Cole Eye Institute, Cleveland Clinic, 9500 Euclid Avenue, Cleveland, OH 44195 USA; 83 Department of Ophthalmology, Duke University Medical Center, Durham, NC USA; 84 Department of Medicine, Duke University Medical Center, Durham, NC USA; 85 Duke Molecular Physiology Institute, Duke University Medical Center, Durham, NC USA; 86 Bascom Palmer Eye Institute, University of Miami Miller School of Medicine, 3880 Tamiami Trail North, Naples, FL 34103 USA; 87 Department of Ophthalmology, Royal Perth Hospital, Perth, Western Australia 6001 Australia; 88 Department of Ophthalmology, NYU School of Medicine, New York, NY USA; 89 Department of Medical Genetics, Cambridge Institute for Medical Research, University of Cambridge, Cambridge, CB2 0QQ UK; 90 Department of Ophthalmology, Cambridge University Hospitals NHS Foundation Trust, Cambridge, CB2 0QQ UK; 91 Department of Ophthalmology UCSF Medical School, San Francisco, USA; 92 Department of Ophthalmology and Visual Sciences, Vanderbilt University, Nashville, Tennessee United States; 93 Casey Eye Institute, Oregon Health & Science University, Portland, OR 97239 USA; 94 Department of Human Genetics, Graduate School of Public Health, University of Pittsburgh, Pittsburgh, PA 15261 USA; 95 School of Clinical Sciences University of Edinburgh, EH16 4SB Scotland, UK; 96 Center for Inherited Disease Research (CIDR) Institute of Genetic Medicine Johns Hopkins University School of Medicine Baltimore, Baltimore, MD USA; 97 Department of Ophthalmology, David Geffen School of Medicine—UCLA, Stein Eye Institute, Los Angeles, CA 90095 USA; 98 Department of Human Genetics, David Geffen School of Medicine—UCLA, Los Angeles, CA 90095 USA; 99 Department of Human Genetics, Radboud University Medical Centre, Nijmegen, the Netherlands; 100 Department of Pathology & Cell Biology, Columbia University, New York, NY USA; 101 Center for Translational Medicine, Moran Eye Center, University of Utah School of Medicine, Salt Lake City, UT USA; 102 Division of Preventive Medicine, Brigham & Women’s Hospital, Harvard Medical School, Boston, USA; 103 Division of Epidemiology and Clinical Applications, Clinical Trials Branch, National Eye Institute, National Institutes of Health, Bethesda, MD 20892 USA; 104 Institute for Computational Biology, Case Western Reserve University School of Medicine, 2103 Cornell Rd, Cleveland, OH 44106 USA

**Keywords:** Computational biology and bioinformatics, Molecular medicine, Pathogenesis, Gene expression, Gene regulation, Genetic association study, Quantitative trait

## Abstract

Genome-wide association studies (GWAS) for late stage age-related macular degeneration (AMD) have identified 52 independent genetic variants with genome-wide significance at 34 genomic loci. Typically, such an approach rarely results in the identification of functional variants implicating a defined gene in the disease process. We now performed a transcriptome-wide association study (TWAS) allowing the prediction of effects of AMD-associated genetic variants on gene expression. The TWAS was based on the genotypes of 16,144 late-stage AMD cases and 17,832 healthy controls, and gene expression was imputed for 27 different human tissues which were obtained from 134 to 421 individuals. A linear regression model including each individuals imputed gene expression data and the respective AMD status identified 106 genes significantly associated to AMD variants in at least one tissue (Q-value < 0.001). Gene enrichment analysis highlighted rather systemic than tissue- or cell-specific processes. Remarkably, 31 of the 106 genes overlapped with significant GWAS signals of other complex traits and diseases, such as neurological or autoimmune conditions. Taken together, our study highlights the fact that expression of genes associated with AMD is not restricted to retinal tissue as could be expected for an eye disease of the posterior pole, but instead is rather ubiquitous suggesting processes underlying AMD pathology to be of systemic nature.

## Introduction

Age-related macular degeneration (AMD) is a frequent disease of the choroid, Bruch’s membrane, retinal pigment epithelium and photoreceptor complex characterized by a progressive loss of vision in older individuals of industrialized countries. For the year 2016, it was estimated that worldwide approximately 162 million were affected by early AMD (prevalence 8.0%) and 10 million people by late AMD (prevalence 0.4%). These numbers were predicted to increase to 260 million patients with early and 19 million with late AMD by the year 2040^1^.

It is well established that AMD is a complex disease, involving environmental and genetic risk factors. The International AMD Genomics Consortium (IAMDGC) performed a genome-wide association study (GWAS) and reported 7,218 genetic variants to be associated with late-stage AMD at a genome-wide significance level (P-value ≤ 5 × 10^−8^). Sequential forward selection finally identified 52 independent association signals, which are distributed over 34 genomic loci^2^. An additional 11,768 variants (P-value ≤ 5 × 10^−4^) failed to reach genome-wide significance in this study but may well play a role in AMD pathogenesis. These variants may become relevant only with an increase in sample-size in future GWAS, or by gathering additional information on the functional impact of these variants in relation to AMD pathology.

Typically, GWAS rarely point to genetic variants with a clear functional impact on cellular integrity, particularly since the majority of genetic variants identified by GWAS are located in non-coding, intronic or intergenic regions of the genome. However, the latter variants in particular may play an important role in regulating gene expression^3^. An attractive approach to overcome such limitations of GWAS is to correlate the disease-association of single variants with mRNA expression in a given tissue utilizing large-scale mRNA expression studies. Such analyses result in data known as expression quantitative trait loci (eQTL)^4^.

Generally, eQTL are calculated in healthy tissues to identify genes whose expression is regulated by genetic variation which, in turn, may be useful to further understand disease etiology based on the concurrence of GWAS signals and genetic variation altering gene expression. At present, two studies explored single tissue eQTL in the context of AMD by investigating liver^5^ and retinal^6^ tissue. These approaches successfully identified eQTL in liver for five AMD loci, and in retina for nine of the 34 AMD-associated loci (Q-value ≤ 0.05). However, for the majority of variants the causative signal remains elusive. Evaluation of local eQTL in the context of complex diseases usually includes only the lead variant of a disease-associated locus as linkage disequilibrium (LD) and haplotype structures complicate the analysis. For AMD, a total of 7,218 variants would need to be investigated, which leads to a high burden for multiple testing potentially obscuring real biological signals.

To overcome the limitations of single eQTL analysis, a promising approach was established and is known as transcriptome wide association study (TWAS). In TWAS more complex models than least squares linear regression are applied. These typically include models used in classical machine learning, such as ridge regression, lasso regression, or elastic net, and aim to determine a set of genetic variants which consistently influence gene expression in a given tissue. In a further step, these variants are extracted from classical GWAS datasets to predict their influence on relative gene expression eventually relevant to disease processes. Thus, correlating imputed gene expression to disease status appears to be appropriate to identify disease-associated genes^7–9^. It should be noted however that TWAS are not suited to extract information on gene expression alterations at the time of disease manifestation as the model building solely includes gene expression determined in healthy tissue. As a major feature, TWAS need less correction for multiple testing than eQTL analysis, mainly due to the fact that the calculations are based on several thousand genes instead of usually seven to twelve million genetic variants. In addition, TWAS involve potential combinatory effects of genetic variants, an invaluable benefit over approaches using simple single genetic variant models.

Gamazon *et al*. (2015) proposed a gene-based association method termed PrediXcan to perform TWAS based on data of the Genotype-Tissue Expression (GTEx) project and respective individual genotype information from GWAS studies^8,10^. The required prediction model weights of up to 48 different tissues can be downloaded through the website PredictDB (http://predictdb.org). TWAS based on individual genotypes identify disease-associated genes in known loci from GWAS, and also include genomic regions, which were initially not disease-associated as they did not reach genome-wide significance. This way, TWAS permit the detection of novel disease-associated genes.

TWAS data derived from various tissues are especially valuable for complex diseases with unknown underlying pathomechanisms. Although AMD pathology appears restricted to the posterior pole of the eye, several studies have highlighted systemic effects of AMD-associated genes^11–15^. To this effect, previous studies revealed a significant association of late-stage AMD with the genetic risk of 16 seemingly unrelated complex traits and diseases including psoriasis, rheumatoid arthritis and systemic lupus erythematosus as well as blood lipid levels^16^.To identify potentially differential expressed genes in AMD cases compared to controls, we performed a TWAS based on the individual level imputed expression data of 33,976 individuals and included models derived from 27 human tissues.

## Results

### Identification of genes associated with AMD genetics

**Figure 1:**
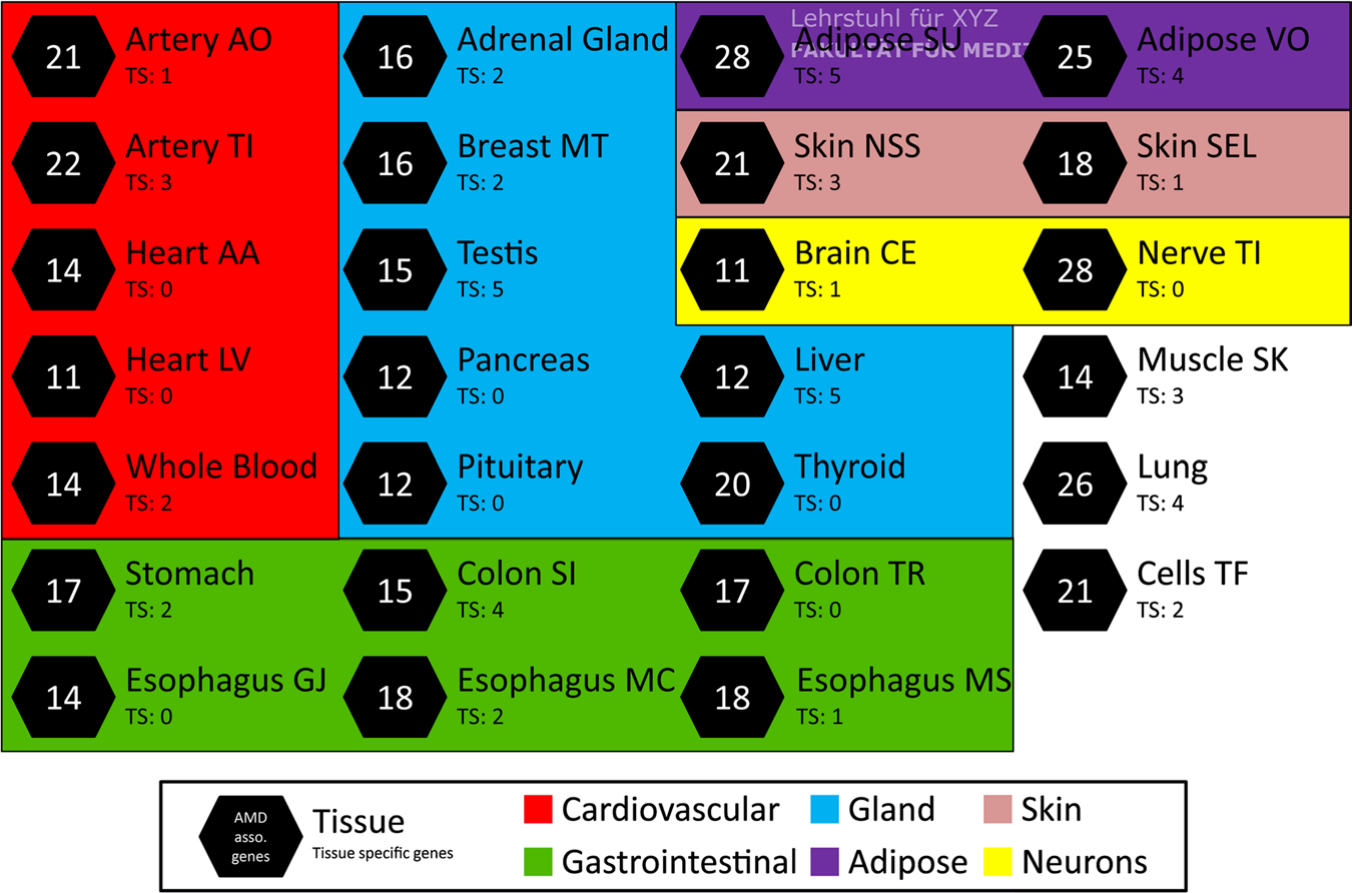
TWAS results for 27 tissues. A TWAS was conducted based on the genotypes of 16,144 late-stage AMD cases and 17,832 AMD-free controls. Prediction models of 27 tissues were included in the analysis. The schematic overview demonstrates the number of significant AMD-associated genes (FDR < 1 × 10^−3^) within the respective tissue. If a gene was found exclusively in a single tissue, it was marked as tissue-specific (TS). Tissue classification was performed manually according to main functions or metabolic assignments. Adipose SU: Adipose Subcutaneous; Adipose VO: Adipose Visceral Omentum; Artery AO: Artery Aorta; Artery TI: Artery Tibial; Brain CE: Brain Cerebellum; Breast MT: Breast Mammary Tissue; Cells TF: Cells Transformed fibroblasts; Colon SI: Colon Sigmoid; Colon TR: Colon Transverse; Esophagus GJ: Esophagus Gastroesophageal Junction; Esophagus MC: Esophagus Mucosa; Esophagus MS: Esophagus Muscularis; Heart AA: Heart Atrial Appendage; Heart LV: Heart Left Ventricle; Muscle SK: Muscle Skeletal; Nerve TI: Nerve Tibial; Skin NSS: Skin Not Sun Exposed Suprapubic; Skin SEL: Skin Sun Exposed Lower leg.

The main objective of this study is to identify potentially relevant genes in AMD etiology. To this end, we conducted a TWAS based on the IAMDGC dataset^2^ and applied the PrediXcan^8^ algorithm based on genotype and phenotype data from 16,144 late-stage AMD cases (including clinical diagnoses of geographic atrophy and/or choroidal neovascularization), and from 17,832 AMD-free controls. The prediction models from 27 tissues were retrieved via PredictDB (number of samples per tissue between 134 and 421) and were implemented into our analysis. We imputed gene expression for each tissue separately and applied a linear regression model to identify late-stage AMD-associated genes based on the AMD status of each individual. After correction for multiple testing, we considered genes with a Q-value smaller than 0.001 to be significantly associated with AMD in the corresponding tissue (Supplementary Fig. 1). This stringent threshold was chosen to avoid false positive results. In each tissue, a minimum of 11 (see “Brain Cerebellum” and “Heart Left Ventricle”) and up to 28 (see “Adipose Subcutaneous” and “Nerve Tibial”) AMD-associated genes (Fig. 1 and Supplementary Table S1a,b) were identified (mean 17.63; SD 5.02). Altogether, 106 unique genes were significantly associated to AMD in at least one tissue. Of these, 88 genes are located in loci that are known to be AMD-associated with genome-wide significance (Table 1 and Supplementary Table S1c)^2^. Moreover, 18 additional genes were not located in proximity (window size of 1MB) to any of the 52 independent hits identified by Fritsche *et al*. (2016), and may represent novel AMD loci (Table 2).

**Table 1:** AMD-associated genes located in known AMD loci.

AMD locus^a^	Locus name^a^	Chr	AMD-associated genes in TWAS analysis^b^ (Number of significant tissues (FDR < 0.001) and effect direction within tissues)^c^
1	*CFH*	1	*CFHR3* (20+); *CFHR1* (14+); *CFH* (10−/1+); *KCNT2* (6−); *ZBTB41* (5+); *CFHR4* (2+); *F13B* (1+); *ASPM* (1+); *RP11.332L8.1* (1−); *DENND1B* (1+); *LHX9* (1−)
2	*COL4A3*	2	*COL4A3* (2−)
4	*COL8A1*	3	*NIT2* (5−); *TBC1D23* (4−); *RP11.114I8.4* (2+); *TOMM70A* (2+); *TMEM45A* (1−)
5	*CFI*	4	*PLA2G12A* (13+); *CASP6* (3+); *CCDC109B* (1−); *CFI* (1−)
9	*VEGFA*	6	*PPP2R5D* (2−)
11	*PILRB/PILRA*	7	*STAG3L5P* (27+); *PILRB* (27+); *PILRA* (26+); *PMS2P1* (14−); *TSC22D4* (8+); *ZCWPW1* (3+); *NYAP1* (3−); *ZKSCAN1* (1−); *STAG3* (1−)
10	*KMT2E/SRPK2*	7	*RP11.325F22.5* (1+); *RP11.325F22.2* (1+)
12	*TNFRSF10A*	8	*TNFRSF10A* (14−)
14	*TRPM3*	9	*TRPM3* (1+)
13	*MIR6130/RORB*	9	*RORB* (1−)
15	*TGFBR1*	9	*TGFBR1* (1+)
18	*ARMS2/HTRA1*	10	*PLEKHA1* (17−/1+); *BTBD16* (11+/ 3−); *ARMS2* (14−); *HTRA1* (5+/ 2−); *DMBT1* (3−); *ATE1* (2+); *RP11.318C4.2* (2−); *FGFR2* (1−); *TACC2* (1−); *RP11.107C16.2* (1−); *RP11.564D11.3* (1+); *IKZF5* (1−); *ACADSB* (1−)
19	*RDH5/CD63*	12	*RDH5* (17−); *BLOC1S1* (1+)
21	*B3GALTL*	13	*B3GALTL* (5+)
22	*RAD51B*	14	*PLEKHH1* (2+)
23	*LIPC*	15	*ALDH1A2* (1+); *LIPC* (1+)
24	*CETP*	16	*CETP* (4−); *HERPUD1* (2−); *NLRC5* (2−); *MT1DP* (1+); *GPR56* (1−)
25	*CTRB2/CTRB1*	16	*CFDP1* (4−); *BCAR1* (2−); *TMEM170A* (2+)
26	*TMEM97/VTN*	17	*TMEM199* (10+); *POLDIP2* (3+); *TMEM97* (2+)
27	*NPLOC4/TSPAN10*	17	*C17orf70* (1+); *NPLOC4* (1+); *PDE6G* (1−)
29	*CNN2*	19	*MED16* (3+); *GRIN3B* (2−); *ABCA7* (2−); *AC006273.5* (1+); *CNN2* (1−)
28	*C3*	19	*GPR108* (22+); *CTC.503J8.6* (1−); *GTF2F1* (1−)
30	*APOE*	19	*RELB* (1−); *BLOC1S3* (1+); *DMPK* (1+)
31	*MMP9*	20	*PLTP* (10+); *SLC12A5* (8−/1+); *SPATA25* (1+); *NEURL2* (1+)
34	*SLC16A8*	22	*BAIAP2L2* (2−); *PICK1* (1−); *CBY1* (1−)

^a^AMD locus number and name according to Fritsche *et al*.^2^. ^b^AMD-associated genes in TWAS analysis overlapping with the locus of Fritsche *et al*. (2016) (ref. ^2^) in a +/− 1 Mb window. ^c^Positive effect direction (+) points to predicted gene expression being higher in AMD cases compared to controls and vice versa for negative effect direction (−). Chr = Chromosome.

**Table 2:** AMD-associated genes located in novel loci that did not reach genome-wide significance in previous GWAS studies^2^.

Gene	Chr	Gene Expression Prediction			Strongest effect tissue^a^	Model Information in Strongest Effect Tissue	
		Predictable Tissues	AMD associated (FDR < 0.001)	Mean effect size (SD)		Variants in model	AMD associated (GWAS P-value < 1 × 10-04)^b^
*C1orf21*	1	15	1	−0.028	Liver	34	3
*CD55*	1	17	3	−0.016 (0.003)	Esophagus Muscularis	59	5
*CR2*	1	3	1	−0.013	Muscle Skeletal	40	6
*NOSTRIN*	2	18	1	−0.015	Esophagus Mucosa	44	0
*PPIL3*	2	27	16	0.037 (0.004)	Adipose Subcutaneous	37	24
*NDUFB3*	2	6	4	0.005 (0.001)	Adipose Subcutaneous	12	8
*ADAM19*	5	21	12	−0.013 (0.006)	Adipose Subcutaneous	25	8
*IP6K3*	6	17	1	0.019	Cells Transformed fibroblasts	22	1
*ZFP37*	9	10	1	−0.017	Adipose Subcutaneous	53	0
*RP11.777F6.3*	11	2	1	0.007	Testis	5	2
*CEP57*	11	23	3	−0.02 (0.005)	Skin Not Sun Exposed Suprapubic	48	0
*AP001877.1*	11	24	8	−0.016 (0.006)	Nerve Tibial	50	0
*RIN3*	14	13	1	0.018	Colon Sigmoid	24	2
*ULK3*	15	17	1	−0.01	Lung	10	4
*USP7*	16	3	1	0.014	Muscle Skeletal	55	1
*FUT2*	19	11	1	−0.009	Lung	23	0
*MAMSTR*	19	9	1	0.007	Adrenal Gland	13	0
*LILRA3*	19	23	1	−0.016	Colon Sigmoid	15	2

^a^Tissue, which showed the highest absolute effect size. ^b^Number of variants in prediction model, which were AMD-associated with a p-value smaller than 1 × 10^−04^ in Fritsche *et al*. (2016) (ref. ^2^). Chr = Chromosome.

Positive effect sizes point to predicted gene expression in healthy tissue being higher in AMD cases than controls, whereas negative effect sizes are indicative of decreased gene expression. The largest effect sizes ranged from −0.38 (*ARMS2*, Testis) to +0.35 (*CFHR1*, Liver). The mean absolute effect size across all AMD-associated genes (Supplementary Table S1b) was 0.035 (SD: 0.039). Four of the 106 genes showed remarkably higher absolute effect sizes in comparison to the remaining genes. These include the CFH-related genes CFHR1, 3 and 4 (positive effect sizes; higher gene expression in cases compared to controls) and ARMS2 (negative effect size; lower gene expression in cases compared to controls). Notably, ARMS2 gene expression is AMD-associated in all 14 predictable tissues, with a mean effect size of −0.098 (SD: 0.09) (Supplementary Table S1c).

Interestingly, 54 of the 106 genes were significantly AMD-associated in more than one of the 27 tissues interrogated. Sixteen genes (*ADAM19*, *ARMS2*, *BTBD16*, *CFH*, *CFHR1*, *CFHR3*, *GPR108*, *PILRA*, *PILRB*, *PLA2G12A*, *PLEKHA1*, *PMS2P1*, *PPIL3*, *RDH5*, *STAG3L5P*, *and TNFRSF10A*) were associated with AMD disease status in over 10 tissues, pointing to effects likely acting in systemic processes. Moreover, the predicted gene expression of three genes (*PILRA*, *PILRB*, and *STAG3L5P*) located within known AMD Locus 11^2^ was significantly AMD-associated in nearly all tissues analyzed (Supplementary Table S1c). A total of 52 out of 106 genes were significantly AMD-associated in only one of the 27 tissues analyzed.

To further validate these findings, we tested the variants used for prediction of gene expression for their genome-wide significant association with AMD status. This analysis was performed for each gene-tissue pair of the 106 AMD-associated genes separately to allow for tissue dependency of the imputation models in gene expression (Supplementary Table S1b). Many of the identified genes, which are located in loci known to be AMD-associated with genome-wide significance^2^, were associated with AMD status in several tissues. To facilitate interpretation, we had a detailed look at the prediction models of the tissues, which showed the highest absolute effect sizes (“strongest effect tissue”) for each of the corresponding 88 genes. We observed that 66 of 88 genes harbor at least one genome-wide significant AMD-GWAS variant (Supplementary Table S1c). Interestingly, only for a single gene (*KCNT2*), all of the prediction model variants are also significantly associated with AMD. For 49 of the 66 genes less than 50% of the prediction model variants were AMD-associated with genome-wide significance. We further investigated if any of the prediction model variants for the 22 remaining genes showed a weak AMD-association signal in the GWAS of Fritsche *et al*. (GWAS P-value < 1 × 10^−04^)^2^. This was the case for 20 genes, leaving *AC006273.5* (“Skin not sun exposed suprapubic”) and *ZKSCAN1* (“Artery aorta”), which did not include any potentially AMD-associated variant (Supplementary Table S1b).

Moreover, we analyzed whether the 18 of 106 AMD-associated genes which are located in novel AMD loci, included variants with a sub-threshold AMD-association signal for gene expression prediction. This was true for 12 genes showing P-values below the threshold for suggestive association in the latest AMD GWAS (GWAS P-value < 1 × 10^−04^)^2^ (Table 2).

### Network analysis of the 106 genes associated with AMD

**Figure 2:**
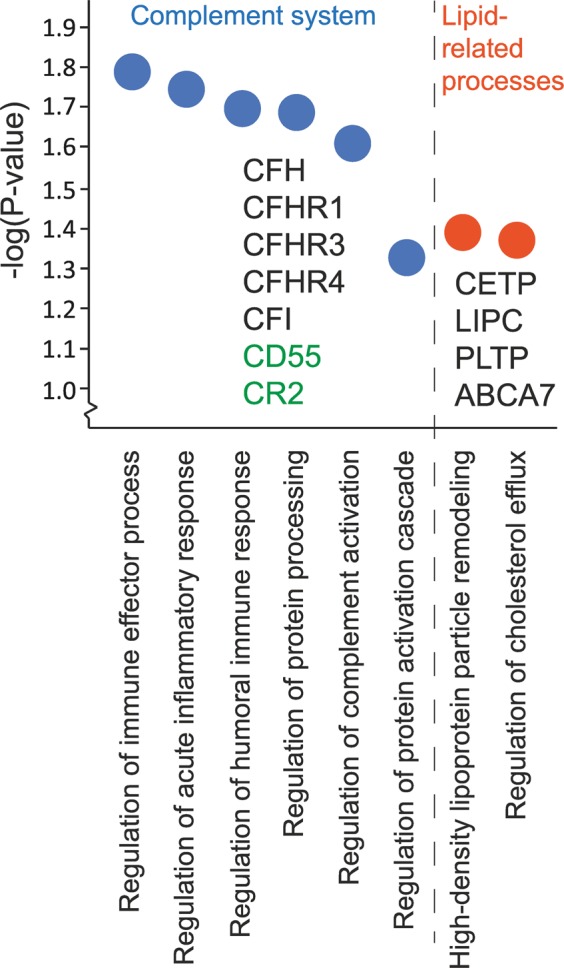
Enriched GO biological processes in 106 genes identified (adjusted P-value < 0.05). Enrichr was used to assign gene ontology (GO) terms for the 106 AMD-associated genes and to investigate enriched GO biological processes^60^. The eight significantly enriched processes are shown, clustered into the complement cascade and lipid-related processes. Genes are given for each process. Genes colored in green indicate those which were not identified previously in AMD-associated loci.

To further explore the biological function of the 106 genes found to be associated with AMD status in this study, we performed an enrichment analysis based on gene ontology (GO) terms (Supplementary Table S2a). For 17 genes, no GO terms were found and no function had been assigned to the genes so far. This latter group contains eight pseudogenes, five long non-coding RNAs, and four protein coding genes (Supplementary Table S2b). The other 89 genes are involved in a variety of biological pathways, of which eight are significantly enriched (adjusted P-value < 0.05). All eight significantly enriched pathways are related to either the complement pathway or to lipid related processes (Fig. 2). Remarkably, we found two novel genes associated with AMD status acting in the complement pathway, namely *CD55* and *CR2*, next to five complement genes in already established AMD loci (*CFH*, *CFHR1*, *CFHR3, CFHR4*, *CFI*)^2^. The enrichment of genes in lipid related processes is reflected by genes such as *CETP*, *LIPC*, *PLTP* and *ABCA7*.

### Comparison to AMD TWAS of retinal tissue

GTEx reveals no data entries for eye tissues. Recently, Ratnapriya *et al*. (2019) calculated eQTL based on 406 retinal tissue samples, the majority of which were from AMD cases and only around 100 samples from controls. Based on these data, they performed a TWAS using the summary statistics of Fritsche *et al*. (2016)^2,6,7^ and identified 31 significantly AMD-associated genes (Q-value ≤ 0.001, genetic model R^2^ ≥ 0.01) (Supplementary Table S3). We compared these with our results from the present study to identify potential retina-specific effects. Nine of the 31 genes from Ratnapriya *et al*. (2019) were located within the MHC locus and thus were omitted from our further comparisons. Another 16 genes were AMD-associated in at least one of the 27 tissues within our study and therefore do not appear to reflect retina-specific effects. Furthermore, most of these 16 genes show the same effect direction in retina as in the other tissues tested in our study. There are two exceptions: (1) In retina, the predicted gene expression of *HTRA1* was significantly lower in AMD cases than controls. This was also the case for the two tissues “Esophagus Mucosa” and “Esophagus Gastroesophageal Junction” in our study. In contrast, predicted *HTRA1* expression was significantly higher in AMD cases than controls in five tissues (see “Thyroid”, “Skin Sun Exposed Lower leg”, “Heart Atrial Appendage”, “Pituitary”, and “Testis” in Supplementary Table S1b). (2) Ratnapriya *et al*. (2019) predict *PLA2G12A* gene expression to be lower in AMD cases compared to controls in retinal tissue. In our study, *PLA2G12A* was predictable in 15 out of 27 tissues and significantly AMD-associated in 13 of these. In each of these 13 tissues gene expression of *PLA2G12A* is predicted to be consistently higher in AMD cases compared to controls.

To summarize the retinal findings, six genes were AMD-associated exclusively in retina, but not in any of the 27 tissues investigated in our study. Among these, the long non-coding RNA *STAG3L5P-PVRIG2P-PILRB* and the uncharacterized gene *RP11-644F5.10* (ENSG00000258311) were not measured within the GTEx dataset and therefore no conclusions can be drawn. The remaining four genes are expressed in several GTEx tissues, but were not AMD-associated in our study. *MEPCE* is a protein coding gene located on chromosome 7, and is known to be a 7SK methylphosphate capping enzyme^17^. Another gene, *RLBP1*, encodes the cellular retinaldehyde-binding protein 1 using 11-cis-retinaldehyde or 11-cis-retinal as physiologic ligands. Two transcripts (*PARP12* and *CTA-228A9.3*) have not been characterized so far.

### AMD-associated genes overlapping pleiotropic loci

**Figure 3:**
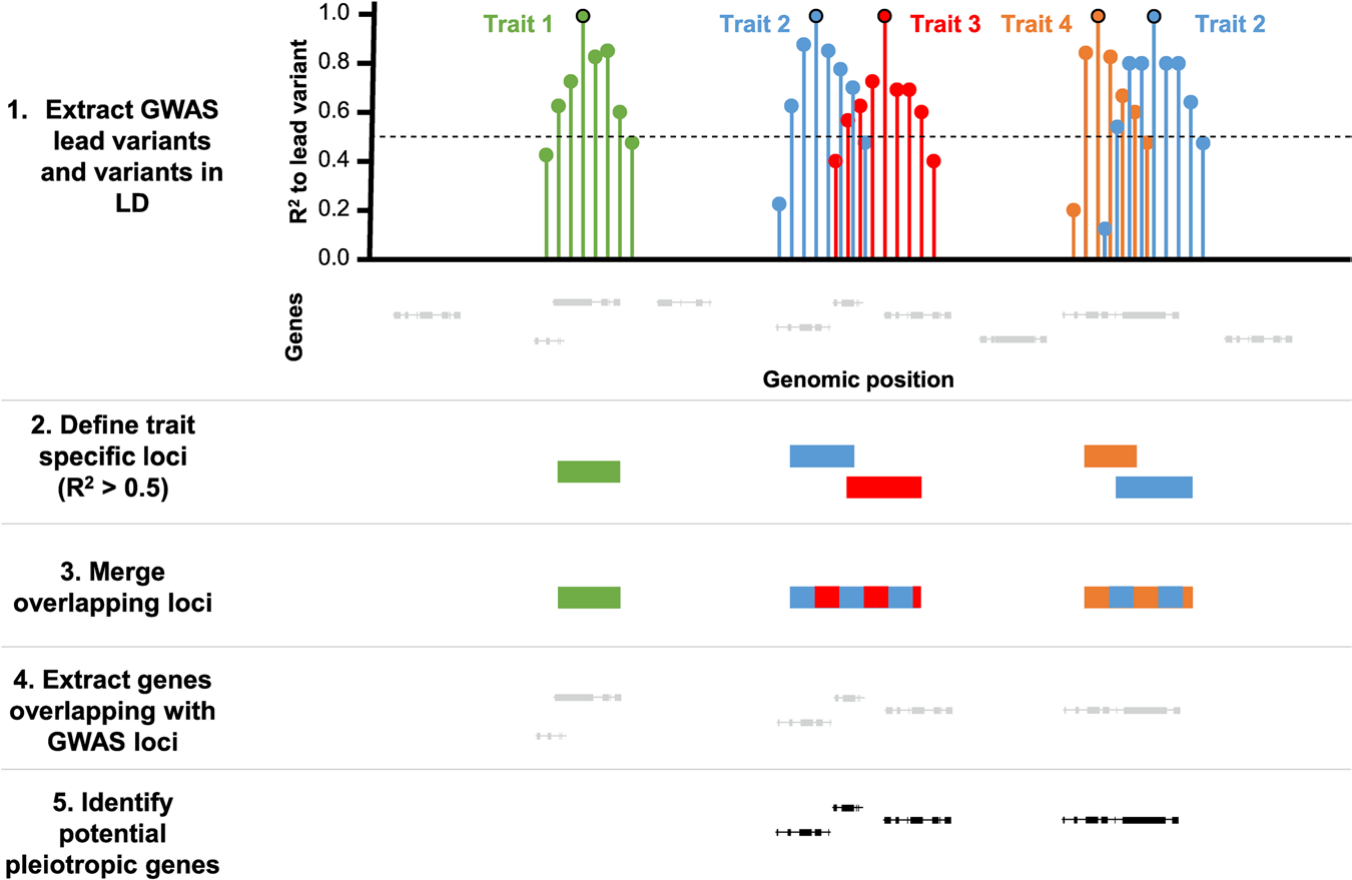
Work-flow for identification of pleiotropic genes. Genome-wide significant independent GWAS signals (P-value ≤ 5 × 10^−8^) were extracted for 82 complex traits and diseases from the corresponding publications. Additionally, variants in LD for each of the independent GWAS hits were included in the analysis. Linked variants with R^2^ > 0.5 demarcated the start- and stop-positions for a GWAS signal R^2^ Locus. Overlapping loci were merged to identify potential pleiotropic genomic regions. The genes overlapping with at least one R^2^ locus were identified.

More than half of the AMD-associated genes identified in this study (54/106) show a significant effect in multiple tissues and are not retina-specific. In addition, our network analysis demonstrates an enrichment of systemic processes such as an involvement in the complement cascade and the lipid metabolism. This raises the question whether AMD-associated genes may also be involved in the pathomechanisms of other complex diseases. We therefore expanded the study of Grassmann *et al*. (2017) and investigated a total of 91 GWAS studies covering 82 complex traits and diseases (Supplementary Table S4a)^16^. First, we extracted the genome-wide significant independent lead variants (P-value ≤ 5 × 10^−8^) for each complex trait or disease and added information about variants in LD from the 1000 G reference data (Fig. 3). Next, we defined R^2^ loci for each GWAS lead variant by summarizing all variants in LD (R^2^ > 0.5). Overlapping loci of different traits were merged to one larger locus. In classical GWAS approaches, genes in direct proximity to the lead variant are *a priori* candidate gene in the sense that such a gene gains high priority in having a functional role in the disease process. For this reason, we identified genes overlapping with R^2^ loci and termed them potentially pleiotropic if the corresponding locus included lead variants of different complex traits and diseases. In a first approach, we added a window of additional 1 Mb up- and downstream to each R^2^ locus in line with Predixcan, which also includes genetic variants within this range around genes^8^. We then merged overlapping 1 Mb loci and determined how many of the 106 AMD-associated genes were located within these loci. Altogether, 105 genes intersected with at least a single 1-Mb-locus. A total of 102 genes were potentially pleiotropic (Supplementary Table S4b). Additionally, 18,813 (77.14%) of the 24,388 predictable genes unique in at least one tissue were also located in these 1-Mb-loci. Such an outcome was due to the extensive size of the constructed loci (mean size: 4286.5 kb; SD: 2778.7) and made result interpretation inaccurate.

**Figure 4:**
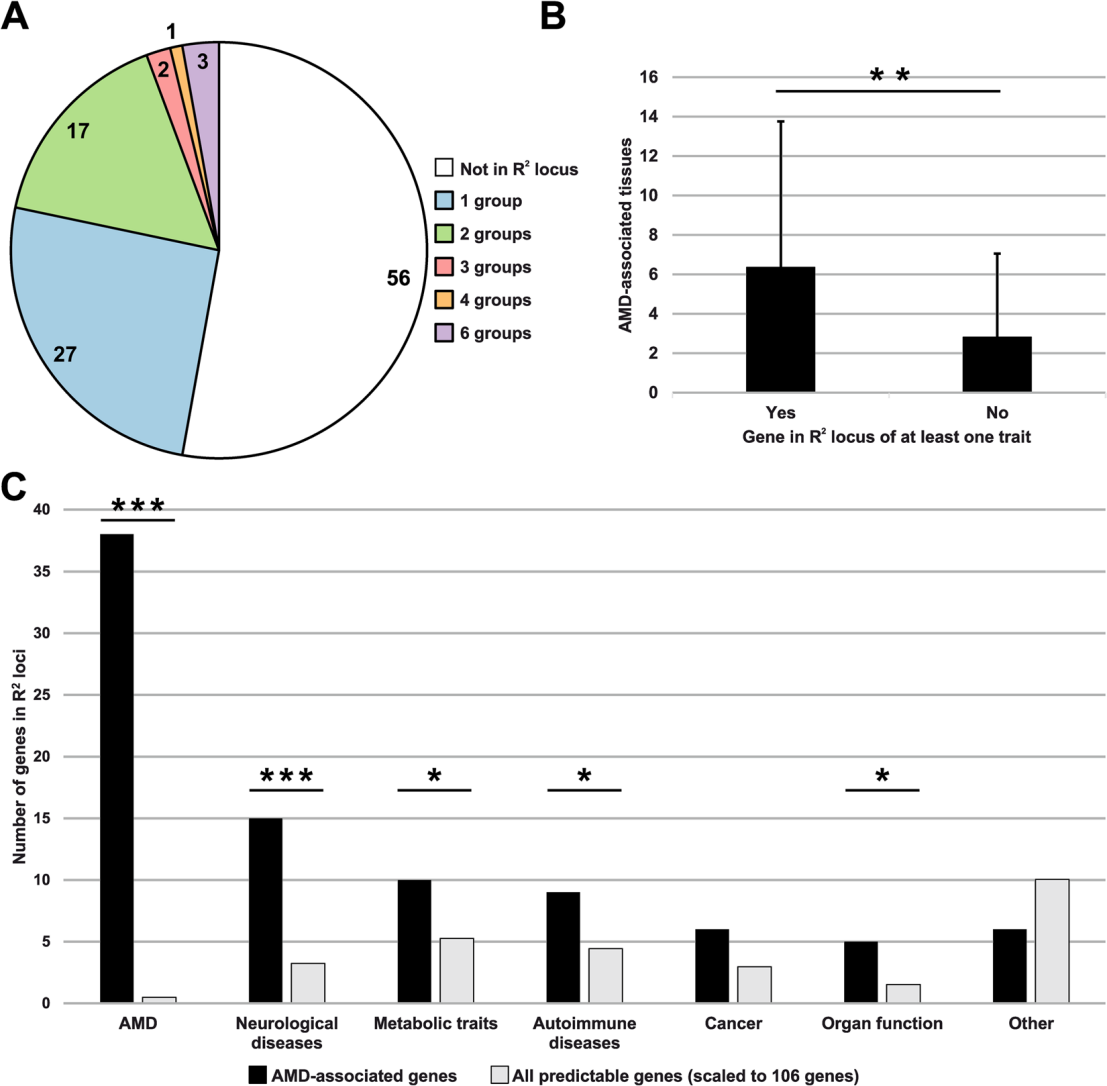
Analysis of AMD-associated genes and their overlap with pleiotropic loci. (**A**) Number of genes, which overlapped with R^2^ loci of trait groups (**B**) Number of AMD-associated tissues per gene as identified by TWAS and overlap of genes with at least a single R^2^ locus (Mann-Whitney-U-Test P-value 0.0053) (**C**) Trait groups shared by AMD-associated genes identified in this study (black) and by all predictable genes (grey). The latter have been scaled from in total 24,388 to 106 genes to enable a better comparability. “Other” include “Aging”, “anthropometric traits”, “Blood cells”, “cardiovascular diseases”, “Complex eye diseases and traits”, “Lifestyle”, and “Immune-related traits”. Significance was assessed through a Fisher exact test. *P-value < 0.05; **P-value < 0.01; ***P-value < 0.001.

We therefore decided to generate R^2^ loci (mean size: 143.1 kb; SD: 208.7) for further analysis. Some of the investigated traits shared variants due to the fact that the traits were exploring the same higher-level pathway as was the case for high-density lipoprotein (HDL) and low-density lipoprotein (LDL). To circumvent this issue, we manually assembled the 82 complex traits and diseases into 13 categories, such as complex eye diseases and traits, AMD, autoimmune diseases, cancer, cardiovascular diseases, metabolic traits, neurological diseases and others (Supplementary Table S4a). A total of 50 AMD-associated genes (47.17%) overlapped with at least one disease group R^2^ locus, including 23 genes, which were potentially pleiotropic (Fig. 4A). Regarding all 24,388 unique predictable genes, 3,846 (15.77%) overlapped with an R^2^ locus. It is important to note that not all of the 88 AMD-associated genes in the reported AMD loci (Table 1) were also located within the R^2^ loci of the AMD category. This is due to variants included into PrediXcan’s prediction models expanding up to one Mb to each side of the start and end of a gene. Interestingly, genes which overlap with at least one R^2^ locus are AMD-associated in more tissues than genes which are not localized within such a locus (Mann-Whitney-U-Test P-value 0.0053) (Fig. 4B). Altogether, the 50 AMD-associated genes which are positioned in a GWAS locus of at least one trait overlap 51 times with a complex trait or disease category outside of AMD, with some genes being linked to multiple groups (Fig. 4C). To test, if this overlap with R2 loci of complex traits or diseases is by chance only, we applied Fisher’s exact test for count data using contingency tables including the 106 AMD-associated genes and the list of all 24,388 unique predictable genes.

Overall, 15 AMD-associated genes overlap with R^2^ loci of neurological diseases (P-Value 7.65 × 10^−7^), 10 genes with metabolic traits (P-Value 0.042) and nine genes with autoimmune diseases (P-Value 0.044). Furthermore, six genes share loci also associated with cancer (P-Value 0.076) and five genes intersect with loci significantly associated to organ function (P-Value 0.018) (Fig. 4C). These data point to systemic and pleiotropic processes in which AMD-associated genes may be involved. Remarkably, only two out of 106 AMD-associated genes are linked to loci of complex eye diseases and traits, namely *RDH5* and *COL4A3*. Almost one quarter (21.7%) of all AMD-associated genes identified in this study overlap with R^2^ loci of two or more complex traits and diseases (Table 3). Three AMD-associated genes (*BCAR1*, *CFDP1*, and *TMEM170A*) residing within a locus on chromosome 16 (chr16:75233867-75516739), coincide with GWAS signals of six different trait groups, namely “organ function”, “cancer”, “neurological diseases”, “autoimmune diseases”, “metabolic traits”, and “AMD”.

**Table 3:** AMD-associated genes overlapping pleiotropic loci.

Gene	Gene Type	AMD associated tissues (FDR < 0.001)	Locus R^2^ [hg19]	Disease groups in locus	Single phenotypes in locus
BCAR1	Protein coding	2	chr16:75233867–75516739	Organ function; Cancer; Neurological diseases; Autoimmune diseases; Metabolic traits; AMD	FEV1FVC; LGF; PanC; MIG; T1D; T2D; AMD
CFDP1	Protein coding	4			
TMEM170A	Protein coding	2			
BAIAP2L2	Protein coding	2	chr22:38295271–38503972; chr22:38505356–38614129	AMD; Anthropometric traits; Metabolic traits; Cancer	AMD; BFP; TG; CMM
ALDH1A2	Protein coding	1	chr15:58671721–58692118; chr15:58718529–58742418	Blood cells; Metabolic traits; AMD	HB; RBC; HDL; TC; TG; AMD
ULK3	Protein coding	1	chr15:75031521–75449869	Cardiovascular diseases; Autoimmune diseases; Blood cells	DBP; GBP; HTN; SBP; SLE; MCV; RBC
COL4A3	Protein coding	2	chr2:228083236–228100488; chr2:228126494–228231432	AMD; Complex eye diseases and traits	AMD; CCT
PMS2P1	pseudogene	14	chr7:99894971–100111776	Neurological diseases; AMD	AD; AMD
STAG3L5P	pseudogene	27			
PILRB	Protein coding	27			
PILRA	Protein coding	26			
ZCWPW1	Protein coding	3			
TSC22D4	Protein coding	8			
NYAP1	Protein coding	3			
RP11.325F22.2	lincRNA	1	chr7:104581402–105063372	AMD; Neurological diseases	AMD; SCZ
PLEKHA1	Protein coding	18	chr10:124124669–124235355	Neurological diseases; AMD	MIG; AMD
ARMS2	Protein coding	14			
HTRA1	Protein coding	7			
RDH5	Protein coding	17	chr12:56115585–56213297	AMD; Complex eye diseases and traits	AMD; MYP
RIN3	Protein coding	1	chr14:93068516–93118229	Cancer; Organ function	BRC; BRConly; FEV1; LGF
CETP	Protein coding	4	chr16:56985514–57006829	AMD; Metabolic traits	AMD; HDL; LDL; TC; TG
FUT2	Protein coding	1	chr19:49158532–49252574	Metabolic traits; Autoimmune diseases	ALP; LEP; TC; CD; IBD; GGT
MAMSTR	Protein coding	1			

AD = Alzheimers disease; ALP = LEP - alkaline phosphatase; AMD = Age-related macular degeneration; BCC = Basal cell carcinoma; BFP = Body fat percentage; BRC = Breast cancer - max. beta; BRConly = Breast cancer - only BRC; CCT = Central corneal thickness; CD = Crohns disease; CMM = Cutaneous malignant melanoma; CSCC = Cutaneous squamous cell carcinoma; DBP = Diastolic blood pressure; eGFR = estimated glomerular filtration rate of creatinine; FEV1 = forced expiratory volume in 1 second; FEV1FVC = forced expiratory volume in 1 second/forced vital capacity; GBP = General blood pressure; GGT = LEP - γ -glutamyl transferase; HB = Haemoglobin; HDL = High-density lipoprotein; HGT = Height; HTN = Hypertension; IBD = Inflammatory bowel disease; LDL = Low-density lipoprotein; LEP = Liver enzymes in plasma; LGF = Lung function; MCV = Mean cell volume; MIG = Migraine; MYP = Myopia; PanC = Pancreatic cancer; RBC = Red blood cell phenotypes; SBP = Systolic blood pressure; SCZ = Schizophrenia; SLE = Systemic lupus erythematosis; T1D = Type 1 diabetes; T2D = Type 2 diabetes; TC = Total cholesterol; TG = Triglycerides.

## Discussion

We performed a TWAS based on the individual data of 16,144 late-stage AMD cases and 17,832 non-AMD controls to further explore causative genes and pathways involved in AMD-associated processes. We predicted gene expression in 27 different tissues and identified 106 genes with significant association to late stage AMD status. An enrichment analysis revealed a significant accumulation of genes involved in the complement cascade and lipid metabolism. For example, gene expression of *CFH* and *CFI*, both regulators of the alternative complement pathway, was found AMD-associated in our study with negative effect sizes suggesting that the expression of these genes is lower in AMD cases than in controls. Consistent with this are earlier findings demonstrating lower complement Factor H (encoded by the *CFH* gene) levels in the sera of AMD patients^18–20^. Furthermore, the expression of negative regulators of *CFH*, specifically *CFHR1*, *CFHR3*, *and CFHR4*, is predicted to be upregulated in AMD cases, which consequently should lead to an increased complement activation.

Our study also identified two genes (*CD55* and *CR2*) in loci, which failed to reach genome-wide significance in previous AMD GWAS^2,21^ Interestingly, the prediction models of both genes included variants which were AMD-associated in the latest AMD GWAS but below the threshold for suggestive association (GWAS P-value < 1 × 10^−04^)^2^ (Table 2). *CD55* and *CR2* are both predicted to be expressed at a lower level in AMD cases compared to controls. CD55 inhibits the C3-convertase and consequently regulates activity of the complement system^22^. Interestingly, homozygous mutations in *CD55*, known to results in loss-of-function, have been found in patients suffering from complement hyperactivation, angiopathic thrombosis, and protein-losing enteropathy (MIM 226300)^23^. While two studies measured *CD55* expression in blood cells of AMD patients, they consistently failed to observe significant differences compared to healthy individuals^24,25^. This is well in line with our current study which predicts *CD55* expression to be AMD-associated exclusively in “Esophagus Muscularis”, “Heart Atrial Appendage”, and “Nerve Tibial”, but not in “Whole Blood”.

CR2 is a member of the complement activation regulator family and plays a role in the humoral immune response^26^. Polymorphisms in *CR2* have been associated with susceptibility to systemic lupus erythematosus type 9 (MIM 610927)^27^. It is important to note that the models used for gene expression prediction in our study were based on normal tissue and therefore likely reveal general effects of gene expression regulation based on the respective genetic background. In general, our findings relating to complement genes support the hypothesis that the complement system is more active in AMD cases than in control individuals. It can be expected that such effects are occurring throughout an entire lifetime well before AMD manifestation. However, gene expression in a diseased tissue may ultimately be unpredictable and significantly different from gene expression in healthy tissue.

The second major finding evident from our list of 106 AMD-associated genes points to lipid metabolism pathways. *LIPC* encodes a protein called hepatic lipase (HL) and is AMD-associated exclusively in liver tissue. HL is secreted into the bloodstream regulating HDL concentration. Lower HL activity was observed to result in higher HDL levels in blood^28^. Our study predicted a higher gene expression of *LIPC* in AMD cases, which then would be expected to result in lower blood HDL levels. This is consistent with an earlier mega-analysis of eQTL in liver tissue which included four independent studies^5^.

Higher levels of HDL were shown in multiple studies to be associated with elevated AMD risk^29–31^. Our data suggest that lower predicted *CETP* expression is significantly associated with AMD in four tissues, but not in liver. As CETP deficiency leads to high HDL levels, this fits earlier findings, that increased HDL is associated with AMD risk^32,33^.

The plasma phospholipid transfer protein (*PLTP*), encoded by another lipid related gene was AMD-associated in 10 out of 27 tissues and showed a mean effect size of 0.017 (SD: 0.006). Interestingly, *PLTP* was not associated with AMD in a previous GWAS but is located in AMD locus 31^2^. PLTP facilitates the transfer of phospholipids and cholesterol in-between lipoproteins. Reduced plasma PLTP activity was shown to cause markedly decreased HDL levels^34,35^. It also plays a role in inflammatory processes and participates in the etiology of atherosclerosis^36,37^. Remarkably, increased PLTP levels in plasma were identified as potential biomarker for AMD in a proteomics-based approach^38^. *ABCA7*, a gene also involved in lipid metabolism, was significantly AMD-associated in our study in lung and whole blood tissue. There is strong evidence that mutations in *ABCA7* are involved in Alzheimer disease (AD-9) (MIM 608907)^39,40^.

Taken together, two major pathways related to AMD pathogenesis, including complement activation and lipid-related processes, were identified through our gene enrichment analysis with both pathways well known in AMD research and thus greatly increasing confidence in the robustness of our data. It is of interest to note that the two pathways are the only significant findings in our study suggesting that the majority (96/106) of AMD-associated genes may function in a plethora of different processes.

As AMD is a disease of the choroid/Bruch’s membrane/retinal pigment epithelium/photoreceptor complex, we conducted a separate TWAS based on retinal tissue^6^. Our initial expectation was to either find an enrichment of AMD-associated genes in the retinal tissue or to even identify a notable number of retina-specific genes implicated in AMD etiology. 16 out of the 22 AMD-associated genes identified in retina by Ratnapriya *et al*.^6^ were also found in at least one non-retinal tissue in our study. This left six genes which potentially harbor a retina-exclusive effect. Remarkably, only one gene, namely *RLBP1*, reveals clear evidence to be causative for a disease of the retina such as Retinitis punctata albescens (MIM 136880) or rod-cone dystrophy (MIM 607476)^41–43^. Nevertheless, *RLBP1* is expressed throughout all tissues of the human body^10^. Interestingly, a recent study regarding schizophrenia, obviously a brain related disease, revealed that 51 (48.1%) of 106 schizophrenia-associated lead variants are eQTL in brain tissue^44^. In contrast, only 9 (17.3%) of the 52 AMD lead variants regulate gene expression in the retina^6^. This observation leads us to the conclusion that changes in retinal gene expression can only partly explain GWAS association signals and that retinal gene expression *per se* is not a suitable criterion to suggest relevance for AMD pathogenesis. Nevertheless, so far no gene expression regulation data of the retinal pigment epithelium (RPE) or the choroid tissue are available. Data about these tissues might enable further conclusions about gene expression regulation at primary sites of AMD pathology.

Genes significantly associated with AMD in a multitude of tissues, like *STAG3L5P*, *PILRB*, *PILRA*, *GPR108*, and *CFHR3*, are likely to act in systemic processes although disease expression appears to be restricted to the posterior pole of the eye. Nevertheless, these genes may affect molecular processes possibly leading to other diseases besides AMD. This is supported by earlier studies associating the genetics of AMD with other complex traits and diseases^16,45^. Here, we analyzed the 106 AMD-associated genes for intersection with pleiotropic genomic regions identified as GWAS R^2^ loci for 82 complex traits and diseases. A striking 50 genes suggested in our study to have relevance to AMD pathology overlap with an R^2^ locus affecting at least one other complex trait or disease. It should be noted that co-localization within a shared genomic locus is not a functional evidence as such, but genes overlapping with a lead variant or a corresponding variant in high LD are *a priori* excellent candidate genes possibly playing a role in disease etiology. Remarkably, 15 genes are located in R^2^ loci of neurological diseases, including 8 genes related to AD, six genes in loci associated to migraine, and one gene overlapping a locus related to schizophrenia.

The findings in AD are of particular interest as AMD and AD share a number of similarities, particularly age-relatedness and pathological deposits in neurological tissue^46^. Correspondingly, beta-amyloid deposits, a hallmark of AD, are also found in retinal deposits of AMD, called drusen^45,47^. Our data point to two AD-related loci which contain AMD-associated genes. One is positioned on chromosome 19 and contains *ABCA7*, which was linked to both diseases in earlier studies^40,48,49^. The other locus is on chromosome 7 and contains several genetically regulated genes which could be important for both diseases. These include the genes *TSC22D4*, *NYAP1*, *PMS2P1*, *STAG3L5P*, *PILRB*, *PILRA*, *and ZCWPW1*. Interestingly, some of these genes have already been investigated in the context of AD^50,51^. Currently, it is not clear how the genes in this locus, which was first identified to be AMD-associated in 2016, contribute to AMD etiology^2^. It should be noted that one and the same locus is named differently in AMD- and AD-related research, namely *PILRB-PILRA* (AMD) or *ZCWPW1* (AD)^2,52^.

Less is known about the six AMD-associated genes overlapping with an R^2^ locus of migraine. Interestingly, *HTRA1* which is intensively investigated in AMD is mutated in CARASIL syndrome (MIM 600142), the latter known to be preceded by migraine^53,54^. Furthermore, 10 AMD-associated genes overlap with R^2^ loci of metabolic traits and 9 genes intersect with GWAS loci involved in autoimmune diseases. Both findings are in line with our gene enrichment analysis and are intensely discussed in current research^11,12,30,55^. Noteworthy, our results do not support the previously identified genetic relationship of AMD and cardiovascular disease (CAD), as only a single AMD-associated gene (*ULK3*) overlaps with an R2 locus of CAD^16,56^. However, this does not preclude the possibility that genetics of AMD and CAD may be linked due to effects other than gene expression regulation. Finally, a remarkable observation is that our analysis identified only two genes, namely *RDH5* and *COL4A3*, which overlap with GWAS loci of complex eye diseases and traits except AMD. Unfortunately, drawing conclusions on the direction of effects remains challenging due to the fact that our findings are based on genomic positions and GWAS signals.

In conclusion, genetically based regulatory effects on gene expression represent a lifetime influence. Our TWAS study identified 106 genes with expression predicted to be AMD-associated in at least one of 27 tissues. The disease-associated expression of genes points to various pathways and mechanisms potentially relevant for AMD etiology and other overlapping complex traits and diseases. Future studies, searching for AMD treatment options or for strategies to prevent AMD, should therefore strongly consider that AMD-associated genetics suggests to alter gene expression throughout the whole body and that these mechanisms are likely involved in a spectrum of other common diseases of mankind.

## Methods

### Study samples and genotype data

The genotypes and phenotypes from 33,976 individuals with European ancestry were retrieved from the IAMDGC consortium (see “Data availability statement”). We included the genotypes from 16,144 late-stage AMD cases, presenting with geographic atrophy and/or choroidal neovascularization, and from 17,832 AMD-free control individuals. Detailed inclusion and exclusion criteria as well as comprehensive information about genotype quality control and imputation procedure are given elsewhere^2^. For gene expression imputation, we used the genotype information of 11,722,957 autosomal genetic variants. As PrediXcan does not accept missing values, genotypes have been transformed to an allele dosage format and missing genotypes of single individuals were filled by the most frequent corresponding genotype.

### TWAS analysis

We used the PrediXcan algorithm to predict gene expression based on genotype information^8^. Gamazon *et al*. provided prediction models (available through PredictDB; http://predictdb.org/) trained on the data of European individuals within the GTEx Version 7 release (GTEx-V7_HapMap-2017-11-29.tar.gz)^57^. For 27 of 48 tissues, genotype and gene expression data of more than 130 individuals (between 134 and 421) were available for prediction model building (Supplementary Table S1a). We applied PrediXcan for each of these 27 tissues to predict individual level gene expression of the IAMDGC cohort. We then used R^58^ to calculate the linear regression of predicted gene expression with AMD and control status. Additionally, we adjusted the model for gender, age and the first two principal components of the genotype PCA performed by Fritsche *et al*^2^. To account for multiple testing, we adjusted the P-values of all 181,536 tests using the false discovery rate (FDR)^59^. Genes located within the major histocompatibility complex (MHC) locus (chr6: 28,477,797–33,448,354, hg19) were excluded from our analysis due to its highly complex association structure. Remaining genes with a Q-value smaller than 0.001 were considered to be significantly AMD-associated.

### Network analysis

Enrichr was used to assign gene ontology (GO) terms to our list of AMD-associated genes and to investigate enriched GO biological processes^60^.

### Identification of pleiotropic loci

We investigated AMD-associated gene intersections with loci, which are known to be associated with other complex traits and diseases. To this end, we searched PubMed (www.pubmed.gov) for GWAS of human traits and diseases, which (1) included primarily individuals of European descent and (2) were published prior to November 2016. Detailed inclusion and exclusion criteria for GWAS studies are given elsewhere^16^. After quality control, we included 82 different traits and diseases into our further analysis (Supplementary Table S4a,c). We extracted the genome-wide significant (P-value ≤ 5 × 10^−8^) and independent GWAS signals and extended them by extracting variants in LD using the 1000 G reference data^61^. The entirety of linked variants (R^2^ > 0.5) was used to define start- and stop-positions for every GWAS signal R^2^-Locus. Next, we merged overlapping loci to identify potential pleiotropic genomic regions. We then extracted all ensemble annotated genes and annotations (version 90)^62^ and mapped them to the beforehand identified trait-associated loci. Each gene was subsequently assigned to the corresponding trait if the genomic position overlapped to the R^2^-Loci.

### Statistical evaluation

All bioinformatical analysis steps were conducted using either the Unix command line or the R programming language. Gene expression imputation based on genotypes of the IAMDGC dataset was performed via PrediXcan. PrediXcan applied LASSO, elastic net and the simple polygenic score based on all tissues of the GTEx project to generate a gene expression prediction model for each gene per tissue. Details about the model building process and the quality control measures are given elsewhere^8^. After gene expression imputation for each of the 27 investigated tissues, we applied a linear regression model to correlate gene expression with AMD status. Thereafter, we adjusted P-values for multiple testing using a stringent FDR setting. The number of investigated genes per tissue and accordingly the number of conducted tests are shown in Supplementary Table S1a. We chose a Q-value threshold of 0.001 to minimize the probability to identify false positive genes. A list of all significantly AMD-associated genes (Q-value < 0.001) is provided in Supplementary Table S1b.

For the network analysis, Enrichr outputs an adjusted p-value to evaluate significance of enriched processes. This is based on a score calculated by multiplying the log p-value computed by the Fisher exact test with the z-score of the deviation from the expected rank by the Fisher exact test^60^.

To determine the significance of the overlap of AMD-associated genes with the R^2^ loci, we applied Fisher’s exact test for count data based on the gene list of interest in comparison to all 24,388 unique predictable genes in at least one tissue outside the MHC locus. This was achieved by creating contingency tables and analyzing them with the *fisher.test* function in R.

### Ethics approval and consent to participate

Twenty six international study groups contributed DNA samples from a total of 33,976 individuals with and without AMD disease (IAMDGC), as previously described^2^. Approval was obtained from each participating site by their respective local ethics review board and informed written consent was obtained from each patient^2^. At each site, the study strictly adhered to the tenets of the Declaration of Helsinki.

### Consent for publication

Not applicable.

## Supplementary information

Supplementary Information.

Supplementary Information2.

Supplementary Information3.

Supplementary Information4.

Supplementary Information5.

## Data Availability

The dataset used in this study was retrieved from the IAMD consortium and compiled information from 16,144 people with late-stage AMD and 17,832 control individuals without AMD. Data permitted for sharing by respective institutional review boards (13,379 late stage AMD subjects and 16.246 AMD-free controls) are available at the database of genotypes and phenotypes (dbGaP)^63^ under the accession number phs001039. Publicly available data from genome-wide association studies (GWAS) of human diseases and traits were extracted from the respective publications. The relevant PubMed identifiers of those publications are listed in Supplementary Table S4a. The prediction models for gene expression imputation are available through PredictDB (http://predictdb.org).
